# Tetrahedral framework nucleic acids alleviate irradiation‐induced salivary gland damage

**DOI:** 10.1111/cpr.13381

**Published:** 2022-12-13

**Authors:** Xueping Xie, Wenjuan Ma, Guo Li, Yuxi Zhan, Li Quan, Xiaoxiao Cai

**Affiliations:** ^1^ State Key Laboratory of Oral Diseases, National Clinical Research Center for Oral Diseases West China Hospital of Stomatology, Sichuan University Chengdu China; ^2^ Department of Oral and Maxillofacial Surgery, The First Affiliated Hospital Zhejiang University School of Medicine Hangzhou China; ^3^ China West Normal University Nanchong China; ^4^ Sichuan Inspection and Testing Center for Dental Devices and Materials Ziyang China

## Abstract

In this study, we investigated the role of tetrahedral framework nucleic acids (tFNAs) in irradiation‐induced salivary gland damage in vitro and in vivo. Irradiation‐damaged submandibular gland cells (SMGCs) were treated with different concentrations of tFNAs. Cell activity was measured by CCK‐8 assay. Cell death was detected by Calcein‐AM/PI double staining. Cell apoptosis was assessed by flow cytometry. The expression of apoptosis proteins and inflammatory cytokines were detected by western blot. Body weight, drinking volume, saliva flow rate and lag time was measured 8 weeks after irradiation. Micromorphological changes of submandibular gland were assessed by haematoxylin–eosin and masson staining. Cell proliferation, apoptosis and microvessel density of submandibular gland were evaluated by immunohistochemical staining. tFNAs could promote cell proliferation, inhibit cell apoptosis of irradiation‐damaged SMGCs and reduce irradiation induced cell death. Mechanism studies revealed that tFNAs inhibited cell apoptosis through regulating the Bcl‐2/Bax/Caspase‐3 signalling pathway and inhibited the release of TNF‐α, IL‐1β and IL‐6 to reduce cell damage caused by inflammation. Animal experiments showed that tFNAs could alleviate irradiation‐induced weight loss, increased water intake, decreased saliva production and prolonged salivation lag time and could ameliorate salivary gland damage. tFNAs have a positive effect on alleviating irradiation‐induced salivary gland damage and might be a promising agent for the treatment of this disease.

## Graphical abstract

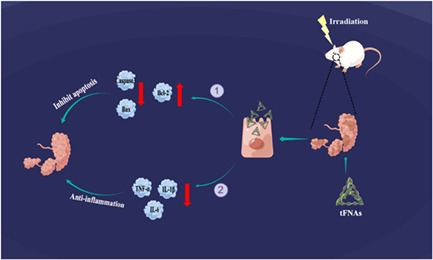

## 1 INTRODUCTION

Radiotherapy is a vital treatment for head and neck cancer. Approximately 80% of patients suffering from head and neck cancer have received radiotherapy. However, radiotherapy can cause many complications such as salivary gland damage, osteonecrosis, mucositis and dental caries, because ionizing radiation tends damage normal tissues surrounding the cancerous tissue.^1^ Among these complications, irradiation‐induced salivary gland damage is the most common, which results in decreased saliva production and can lead to a series of oral problems.^2^, ^3^ This not only reduces the patients' living quality but also seriously affects the course of radiotherapy.^4^, ^5^ At present, the main treatment for irradiation‐induced salivary gland damage is palliative treatment including artificial saliva, saliva substitutes, saliva stimulating agents (pilocarpine), acupuncture therapy, electrostimulation and hyperbaric oxygen therapy.^6^ However, all these methods are only effective for a short time and cannot restore damaged salivary gland tissue; thus, the therapeutic effect of these methods is not satisfactory.^7^ Radioprotectors can reduce irradiation‐induced damage to cells. Amifostine is currently the only radioprotector available. However, its clinical application was limited by its high cost and adverse reactions.^8^, ^9^, ^10^ Many patients receiving chemotherapy and radiotherapy at the same time have great difficulty tolerating these adverse reactions.^11^

Based on the severe situation, research on the treatment of irradiation‐induced salivary gland damage has received extensive attention.^12^, ^13^, ^14^, ^15^ The occurrence of irradiation‐induced salivary gland damage is a complex process involving many factors. In the early stage of radiotherapy, no obvious morphological changes were observed in the salivary glands. The reasons for decreased salivary production include (1) free radicals produced by irradiation damage cell membrane; (2) signalling pathways associated with salivary production, such as calcium signalling, are abnormal; and (3) ion channels or transporters, such as aquaporin 5, are dysfunctional. As radiotherapy is continued, acinar cell apoptosis and acinar become atrophied and ducts dilated. At the same time, a large number of oxygen free radicals can induce cells to produce inflammatory chemokines, which further damage acinus, ducts, blood vessels and nerves.^16^, ^17^, ^18^ In recent years, studies have shown that microvascular endothelial cells in salivary glands are important targets of irradiation injury.^16^, ^19^ It is very important to explore effective methods to restore the damaged salivary gland tissue in this condition.

Tetrahedral framework nucleic acids (tFNAs) have a regular tetrahedral space structure and are a novel nucleic acid nanomaterial assembled by four single DNA strands.^20^, ^21^ tFNAs are favoured by various fields of biomedicine.^22^, ^23^, ^24^, ^25^ Previous studies have demonstrated that tFNAs can promote cell proliferation, migration and differentiation and can inhibit cell apoptosis, which are favourable effects in the field of tissue regeneration.^26^, ^27^, ^28^, ^29^ Moreover, tFNAs can inhibit the release of oxygen free radicals by regulation expression of antioxidative enzyme oxygenase‐1 and can inhibit the release of inflammatory cytokines via inhibiting phosphorylation of mitogen‐activated protein kinase subfamilies.^30^ Thus, tFNAs might serve as a novel antioxidant and anti‐inflammatory agents. Another of her studies showed that tFNAs exhibited protective effects of cells mainly by relieving oxidative stress and decreased kidney injuries after injection in AKI models, which confirmed the anti‐antioxidant efficiency of tFNAs.^31^ Zhou et al.^32^ in 2020 proved that tFNAs treatment could alleviate inflammation both in vitro and in model animals with periodontitis. Wang et al.^33^ utilized tFNAs to treat severe acute pancreatitis in mice for the first time and proved tFNAs to be effective in suppressing inflammation and preventing pathological cell death. Other studies have shown that tFNAs could promote angiogenesis in certain disease models.^34^, ^35^ Altogether, tFNAs exhibit the properties of anti‐apoptosis, anti‐oxidation, anti‐inflammatory and pro‐angiogenesis. We hypothesized that tFNAs could alleviate irradiation‐induced salivary gland damage.

In this study, we investigated whether tFNAs could protect salivary glands from irradiation‐induced damage. Of all salivary glands, pure serous gland (parotid gland) and mixed gland dominated by serous acinus (submandibular gland [SMG]) are most sensitive to irradiation. Usually, about 60%–65% of saliva is produced by the SMG.^36^ Thus, damage to the SMG is more likely to cause serious problems.^37^ We selected the SMG as the study object and tested the role of tFNAs in proliferation and apoptosis of irradiation‐damaged SMGCs. The underlying mechanisms were analysed and explained using detection apoptosis proteins (Caspase 3, Bcl‐2 and Bax) and inflammatory cytokines (TNF‐α, IL‐6 and IL‐1β). Furthermore, we carried out animal experiments to test the repairing effect of tFNAs in irradiation‐induced salivary gland damage.

## 2 MATERIALS AND METHODS

### 2.1 Preparation and Characterization of tFNAs

Synthesis of tFNAs was based on the methods previously reported. TM buffer was consisted of 10 mM Tris‐HCl, pH 8.0 and 50 mM MgCl_2_. Four single‐stranded DNA (ssDNA; Table 1) at an equimolar ratio were added into TM buffer. The mixed solution was heated to 95°C for 10 min, then rapidly cooled to 4°C for 20 min. Construction of tFNAs was verified via 8% sodium dodecyl sulfate polyacrylamide gel electrophoresis (SDS‐PAGE) and capillary electrophoresis. The shape and size of tFNAs was measured by transmission electron microscope (TEM). The hydrodynamic size of tFNAs was measured via dynamic light scattering (DLS) using a Zetasizer Nano‐ZS (Malvern Instruments, England).

**TABLE 1:** Sequence of each single‐stranded DNA (ssDNA).

ssDNA	Direction	Sequence
S1	5′ → 3′	ATTTATCACCCGCCATAGTAGACGTATCACCAGGCAGTTGAGACGAACATTCCTAAGTCTGAA
S2	5′ → 3′	ACATGCGAGGGTCCAATACCGACGATTACAGCTTGCTACACGATTCAGACTTAGGAATGTTCG
S3	5′ → 3′	ACTACTATGGCGGGTGATAAAACGTGTAGCAAGCTGTAATCGACGGGAAGAGCATGCCCATCC
S4	5′ → 3′	ACGGTATTGGACCCTCGCATGACTCAACTGCCTGGTGATACGAGGATGGGCATGCTCTTCCCG

### 2.2 Culture of SMGCs

The SMGCs extraction process was approved by the Ethics Committee. We extracted SMGCs from the SMG of 7‐day‐old SD rats. Specifically, the obtained SMGs were placed in phosphate buffered saline (PBS) containing penicillin–streptomycin solution under aseptic conditions and the capsule, fat, blood vessels and connective tissues were removed. Subsequently, the cleaned SMGs were cut to a size of 1 mm^3^ and digested using the 1:1 mixture of trypsin and type I collagenase for 20 min at 37°C. This step was repeated three to five times. The mixture was centrifuged at 1500 rpm for 5 min. The cell precipitates were resuspended in DMEM/F12 containing 10% FBS, 1% penicillin–streptomycin solution, 10 μg/L EGF, 5 mg/L insulin, 5 mg/L transferrin and 100 μg/L dexamethasone. The cells were cultured in a 37°C incubator containing 5% CO_2_, and the medium was changed every 2 days. Cells in the third passage were used for the experiment.

### 2.3 Cellular uptake of tFNAs

SMGCs with a cell count of 1 × 10^6^ (2 ml) were seeded into a 6‐well plate for 24 h. Cell medium was replaced by serum free medium and serum free medium containing 125 nM Cy5 ligated tFNAs (Cy5‐tFNAs), respectively. 4 h later, cellular uptake of tFNAs was detected by flow cytometry.

### 2.4 Cytotoxicity of tFNAs

SMGCs with a cell count of 5 × 10^3^ (100 μl) were seeded into a 96‐well plate. 24 h later, the medium was changed to fresh medium without FBS but containing tFNAs at gradient concentrations of 62.5, 125, 250 and 375 nM. After incubation for 24 and 48 h, the cell activity was detected by CCK‐8 assay.

### 2.5 Cell proliferation

For the irradiation experiment, SMGCs with a cell count of 5 × 10^3^ (100 μl) were seeded into a 96‐cell plate and cultured at 37°C for 24 h. Cells were irradiated with 20 Gy by a 160 KV x‐ray from a linear accelerator (RS2000, Radsource Technologies Asia Limited). At 24 h after irradiation, cell media were replaced by serum free medium with tFNAs (0, 62.5, 125, 250 and 375 nM). After changing the medium, cells were incubated for 48 h. Cell activity was measured by CCK‐8 assay. Normal cells without any treatment were negative control.

### 2.6 Live and dead cell staining assay

Cells were seeded to a 6‐well plate at a density of 1 × 10^6^/ml and divided into three groups: normal group, irradiated group and tFNAs‐treated group. At 24 h after irradiation, cell medium in the three groups were replaced by serum free medium with or without tFNAs. After changing the medium, cells were incubated for 48 h. The live and dead cells were stained by Calcein‐AM/PI double staining kit. Specially, cells were digested by trypsin without EDTA and centrifuged at 1000 rpm for 5 min. After gently washed twice with PBS, cells were incubated with 200 μl Calcein‐AM working solution followed by 200 μl PI working solution in the dark at room temperature for 15–30 min. Then, cells were rinsed by PBS and detected by fluorescence microscope (Olympus, Japan). The ratio of dead and live cells was quantified by Image J.

### 2.7 Cell apoptosis

At 24 h after irradiation, cell medium in the three groups were replaced by serum free medium with or without tFNAs. After changing the medium, cells were incubated for 48 h. Flow cytometry was used to detect cell apoptosis. Specifically, cells were digested by trypsin without EDTA and centrifuged at 1000 rpm for 5 min. Cell precipitates were resuspended in 400 μl 1 × binding buffer. Then, 5 μl Annexin V and 5 μl PI were mixed with the cell suspension. These were reacted in a dark at room temperature for 5–15 min and detected by flow cytometer at 1 h.

### 2.8 Western blotting

After irradiation and treatment with 125 nM tFNAs, cells were washed by ice‐cold PBS. Total cell proteins were extracted using lysis buffer containing phenylmethanesulfonyl fluoride, phosphatase inhibitor and proteinase inhibitor. 5 × loading buffer was added to each obtained protein sample and mixed well. Then, the mixtures were boiled for 4 min. Those protein samples were separated by 10% SDS‐PAGE followed by transferred to a polyvinylidene fluoride membrane. The protein bands were incubated with primary antibodies to C‐Caspase 3 (Abcam, Cambridge, UK), Caspase 3 (Abcam), Bcl‐2 (Abcam), Bax (Abcam), TNF‐α (Abcam), IL‐1β (Abcam) and IL‐6 (Abcam) at 4°C for 24 h. On the next day, secondary antibodies (Beyotime, Shanghai, China) were added to cover the samples and incubated at room temperature for 1 h. The protein bands were exposed using an enhanced chemiluminescence detection system (Bio‐Rad, Hercules, California). GAPDH was used as the internal control.

### 2.9 In vivo irradiation and tFNAs administration

Animal experiments were approved by the Ethics Committee. SD rats (*n* = 30, 8 weeks; Chengdu Dashuo Life Science and Technology Co., Ltd) were randomly divided into three groups: control (normal rats), IR (PBS‐injected irradiated rats) and IR + tFNAs (tFNAs‐treated irradiated rats). The rats were housed in suspended plastic cages at 22°C–24°C and 50%–60% humidity on a 12‐h light/dark cycle. Food and water were provided ad libitum. Then, food and water were withdrawn for 12 h before irradiation. Animals were anaesthetized with 10% chloral‐hydrate and placed supine in a lead box with only the neck area exposed. To cause damage to the SMG, irradiation was conducted with 160 KV x‐ray emitted from a linear accelerator (RS2000, Radsource Technologies Asia Limited), with a single dose of 15 Gy at a focus‐to‐skin distance of 60 cm. Rats in the control group were anaesthetised but not irradiated. Immediately after exposure, animals were removed from the lead box and housed (5 animals/cage) in the previous environment and allowed free access to food and water. On the second day after irradiation, the rats in the three groups were injected 100 μl PBS, 100 μl PBS and 100 μl 125 nM tFNAs via tail vein, respectively, once every 2 days for 3 weeks.

### 2.10 Biodistribution

To investigate of the biodistribution and SMG target of tFNAs, tFNAs (100 μl, 250 nΜ) was intravenously injected to normal rats and irradiated rats. Then, the rats were euthanized at different time points (15, 45 and 90 min) post‐injection, and SMGs and major organs were harvested and analysed by whole‐body fluorescent system (IVIS, Lumina III Series, PerkinElmer, USA).

### 2.11 Morphological and functional evaluation

#### 2.11.1 Body weight and drinking volume

At Week 8 after irradiation, rats were humane euthanasia. Then, body weights were measured. The drinking volume of mice in each group was also measured at Week 8 after irradiation. Briefly, a total 150 ml of drinking water was added to the bottle. The bottle was inserted into the cage; the amount of water remaining in the bottle at the same time the next day was measured. Average drinking volume = (water added − water remaining)/number of rat.

#### 2.11.2 Saliva flow rate and lag time

Saliva flow rate (SFR) and lag time of salivation were measured at Week 8 after irradiation to evaluate salivary secretory function. After fasted for 12 h, rats were anaesthetised with 10% chloral hydrate, and placed on the experimental table with the head lower than the body. Then, 0.2% pilocarpine was subcutaneously injected into the neck with a dose of 2 mg/kg. A dry cotton ball was used to wipe off saliva, and the cotton ball was discarded. Then, 1.5 ml EP was placed in the corner of the mouth. Saliva was collected for 1 min, weighed, and the saliva flow rate was calculated. Salivation lag time was recorded as the time interval from stimulation to salivation in each group.

#### 2.11.3 Haematoxylin–eosin and masson staining

At week 8 after irradiation, SMGs were separated and fixed in 4% paraformaldehyde for 24 h. All tissues were dehydrated by gradient alcohol and embedded in paraffin. After tissue sections were deparaffinized and rehydrated, we performed haematoxylin–eosin (HE) and Masson staining to observe acinar atrophy and tissue fibrosis. Finally, images of HE and Masson staining were captured by a microscope (Leica).

### 2.12 Immunohistochemistry

#### 2.12.1 Cell proliferation

Anti‐proliferating cell nuclear antigen (PCNA) immunohistochemical staining was used to detect proliferating cells. Tissue sections were deparaffinized and rehydrated, then antigen repaired with citric acid antigen repair solution (pH = 6.0) and heated by microwave with maximum power (98°C–100°C) to boiling and cooled to room temperature; this was repeated twice. 5% BSA was used to block tissue sections at room temperature for 20 min. Then, primary mouse PCNA antibody was added to blocked tissue sections and overnight at 4°C. The next day, primary antibody was removed, and secondary antibody was added and incubated for 1 h. DAB chromogenic solution was prepared, and the tissue sections were covered with DAB chromogenic solution. When a light brown background appeared under the microscope, the chromogenic solution was stopped, and tissue sections were rinsed three times with PBS. Tissue sections was re‐stained with haematoxylin for 2 min and returned to blue with ammonia. Finally, the images were captured by a microscope (Leica). Immunohistochemical results were analysed by ImageJ.

#### 2.12.2 Apoptosis

Apoptotic cells in SMGs were stained by a TUNEL apoptosis detection kit. Tissue sections were deparaffinized and rehydrated, then incubated with a TUNEL reaction mixture for 1 h at 37°C. Then, the tissues were covered by DAB chromogenic solution at room temperature. When a light brown background appeared under the microscope, the chromogenic solution was stopped and the tissues were rinsed three times with PBS. Tissue sections was re‐stained with haematoxylin for 2 min and returned to blue with ammonia. Finally, the images were captured by a microscope (Leica). Immunohistochemical results were analysed by ImageJ.

#### 2.12.3 Microvessel density

Primary CD31 antibody was added to tissue sections and incubated overnight at 4°C. The next steps were the same as described above in the cell proliferation section.

### 2.13 Statistical analysis

All data were statistically analysed using Graphpad Prism Version 9.0.0 (Graphpad Software Inc., USA). Quantitative results were expressed as mean ± SD. The number of repetitions (*n*) and the statistical analysis method are given in the respective figure legends. A *p* < 0.05 was considered statistically significant.

## 3 RESULTS AND DISCUSSION

### 3.1 Characterization of tFNAs

**FIGURE 1:**
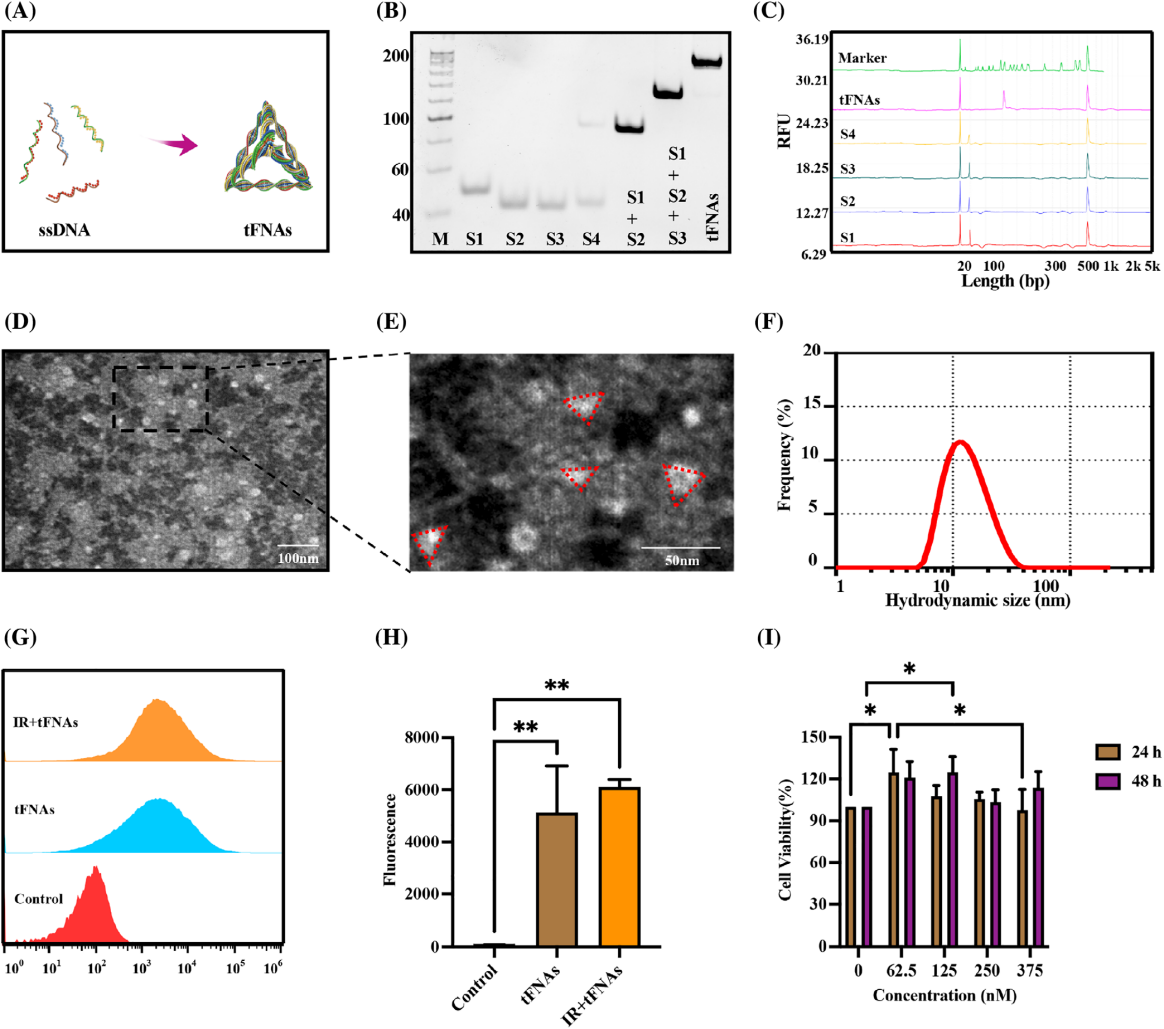
Characterization of tetrahedral framework nucleic acids (tFNAs), cellular uptake and cytotoxicity. (A) Sketch map of tFNAs. (B) Confirmation of the successful synthesis of tFNAs by native sodium dodecyl sulfate polyacrylamide gel electrophoresis. (C) The result of capillary electrophoresis. (D) tFNAs observed by transmission electron microscope (TEM). (E) Local magnification of TEM image. (F) The hydrodynamic size of tFNAs measured by dynamic light scattering. (G) Cellular uptake of tFNAs measured by flow cytometry. (H) Statistical analysis of cellular uptake of tFNAs measured by flow cytometry. Data were presented as mean ± SD, *n* = 3, one‐way ANOVA and Tukey multiple comparisons was used to calculate *p*‐value, **p < 0.01. (I) Cell viability of submandibular gland cells treated with tFNAs at different concentrations. Data were presented as mean ± SD, *n* = 3, two‐way ANOVA and Tukey multiple comparisons was used to calculate *p*‐value, *p < 0.05.

According to Figure 1A, after the solution was heated to 95°C for 10 min and rapidly cooled to 4°C for 20 min, four ssDNA self‐assembled to form tFNAs. 8% SDS‐PAGE was used to check for the successful synthesis of tFNAs. As shown in Figure 1B, the molecular weight of tFNAs was approximately 180 bp, which was the sum of the molecular weights of the four single strands. The nucleic acids migration was detected by capillary electrophoresis (Figure 1C). The peak of tFNAs shifted to the right compared with ssDNA and the molecular weights of tFNAs was around 180 bp which was consistent with the result of SDS‐PAGE. In the TEM image, we observed these particles were roughly triangular (Figure 1D,E). The particle size of tFNAs was determined by DLS. As shown in Figure 1F, the particle size of each tFNA is ~18 nm.

### 3.2 Cellular uptake of tFNAs

SMGCs and irradiated SMGCs were incubated with 125 nM Cy5‐tFNAs for 4 h. The control group was SMGCs incubated with serum free media. We measured cellular uptake of tFNAs by flow cytometry. tFNAs could enter SMGCs and irradiated SMGCs in large numbers (Figure 1G). Fluorescence intensity in SMGCs and irradiated SMGCs were dramatically increased (*p* < 0.01, *p* < 0.01; Figure 1H), and that in irradiated SMGCs was stronger than that in SMGCs although there was no statistical difference. Therefore, tFNAs could enter cells in large quantities, laying a foundation for preforming biological activities.

### 3.3 tFNAs is not cytotoxic to SMGCs

To examine the cytotoxicity of tFNAs, a series of tFNAs at concentrations of 62.5, 125, 250 and 375 nM were incubated with SMGCs. We found that 62.5 nM tFNAs promoted the proliferation of SMGCs at 24 h (*p* < 0.05); 125 nM, 250 nM and 375 nM tFNAs had no significant effect on SMGC proliferation at 24 h. After 48 h, 125 nM tFNAs had a more obvious proliferative effect on SMGCs, the cell activity was increased by 1.27 folds compared with the control group (*p* < 0.05), whereas other concentrations had no effect on cell activity (Figure 1I). These results revealed that tFNAs could promote cell proliferation of SMGCs and had no obvious cytotoxicity.

### 3.4 Effects of tFNAs on cell proliferation of irradiation damaged SMGCs

**FIGURE 2:**
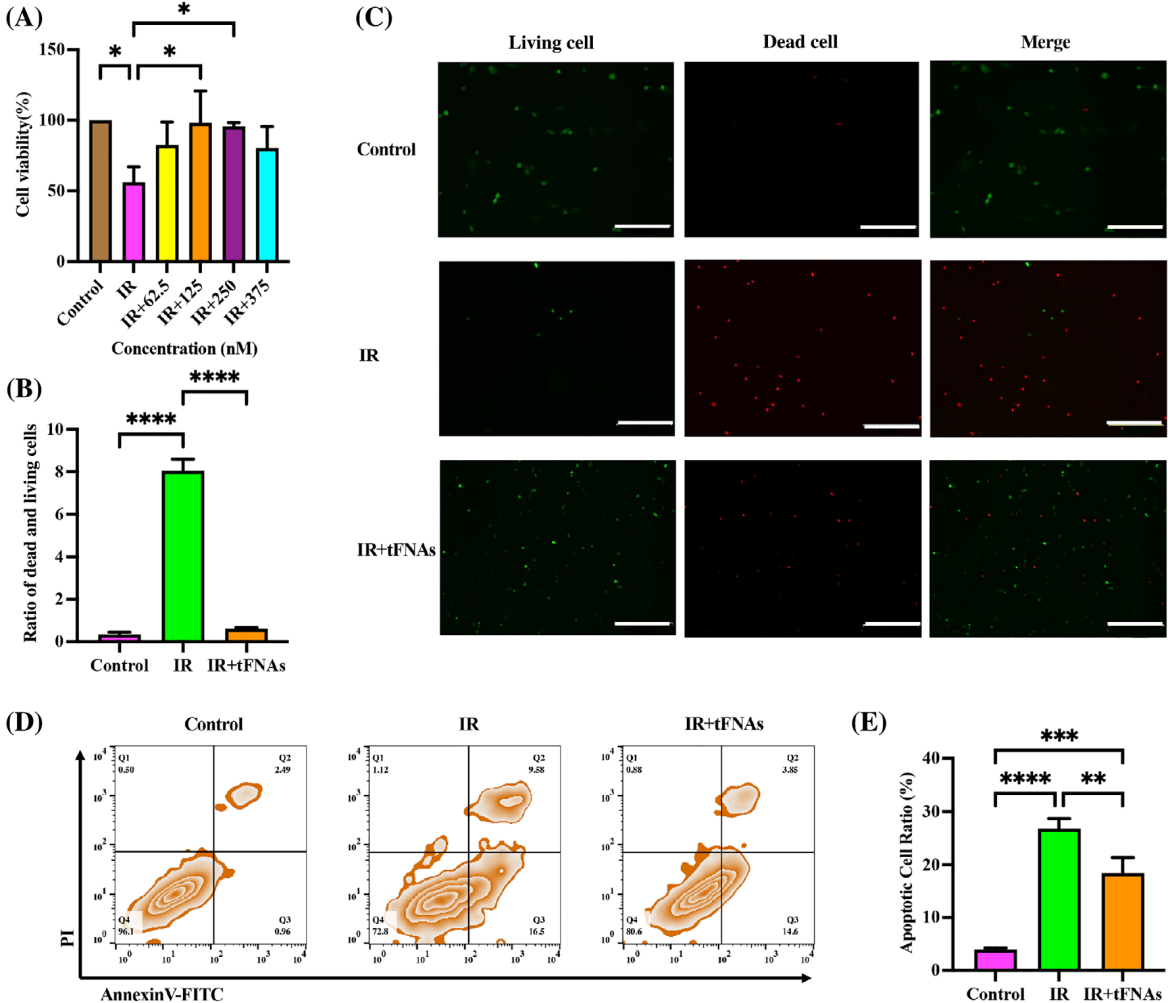
Protected effect of tetrahedral framework nucleic acids (tFNAs) against irradiation induced submandibular gland cells (SMGCs) damage. (A) Cell viability of irradiation damaged SMGCs treated with tFNAs at different concentrations. (Β) Τhe ratio of dead and living cells analysed by Image J. (C) The live and dead cells observed by Fluorescence microscope. Scare bar = 500 μm. (D) Cell apoptosis of normal SMGCs, irradiated SMGCs and tFNAs‐treated irradiated SMGCs measured by flow cytometry. (E) Quantitative analysis of cell apoptosis measured by flow cytometry. Data were presented as mean ± SD, *n* = 3, one‐way ANOVA and Tukey multiple comparisons was used to calculate *p*‐value, *p < 0.05, **p < 0.01, ***p < 0.001, ****p < 0.0001.

At 24 h after irradiation, culture media was changed to serum‐free media containing gradient concentrations of tFNAs (62.5, 125, 250 and 375 nM) and further cultured for 48 h. Then, we used CCK‐8 kit to exam cell activity. When compared with irradiated SMGCs which were irradiated only, the cell activity of tFNAs‐treated irradiated SMGCs was increased; the results of 125 and 250 nM tFNAs were significant and 125 nM should be the optimum concentration (*p* < 0.05, *p* < 0.05; Figure 2A). We chose 125 nM tFNAs for the following experiments.

### 3.5 tFNAs reduce irradiation‐induced cell death

Calcein‐AM/PI double staining was used to detect the living and dead cells. Normal cells in the control group were almost all living cells. The irradiation induced high death rates, whereas the number of death cells decreased after cells incubated with 125 nM tFNAs for 48 h (Figure 2C). The ratio of dead and living cells was analysed by Image J. The result was shown in Figure 2B. The ratio of dead and living cells in the IR group was 22.97‐fold higher than that in Control group, after tFNAs treatment the ratio was decreased significantly which was ~0.075 folds compared with the IR group (Figure 2B). Thus, tFNAs could reduce irradiation‐induced cell death.

### 3.6 Effects of tFNAs on cell apoptosis of irradiation‐damaged SMGCs

Then, we used flow cytometry to exam cell apoptosis. The proportion of early and late apoptotic cells in normal SMGCs was about 3.45% and increased to 26.08% in irradiated SMGCs but decreased to 18.45% when irradiated cells were exposed to 125 nM tFNAs (Figure 2D). tFNAs could significantly reduce irradiation‐induced cell apoptosis (*p* < 0.01 vs. irradiation group, Figure 2E). These results revealed that tFNAs could promote proliferation and inhibit apoptosis of irradiation‐damaged SMGCs, which indicated that tFNAs could protect SMGCs from irradiation damage.

### 3.7 tFNAs inhibit Bcl‐2/Bax/Caspase‐3 signal pathway

**FIGURE 3:**
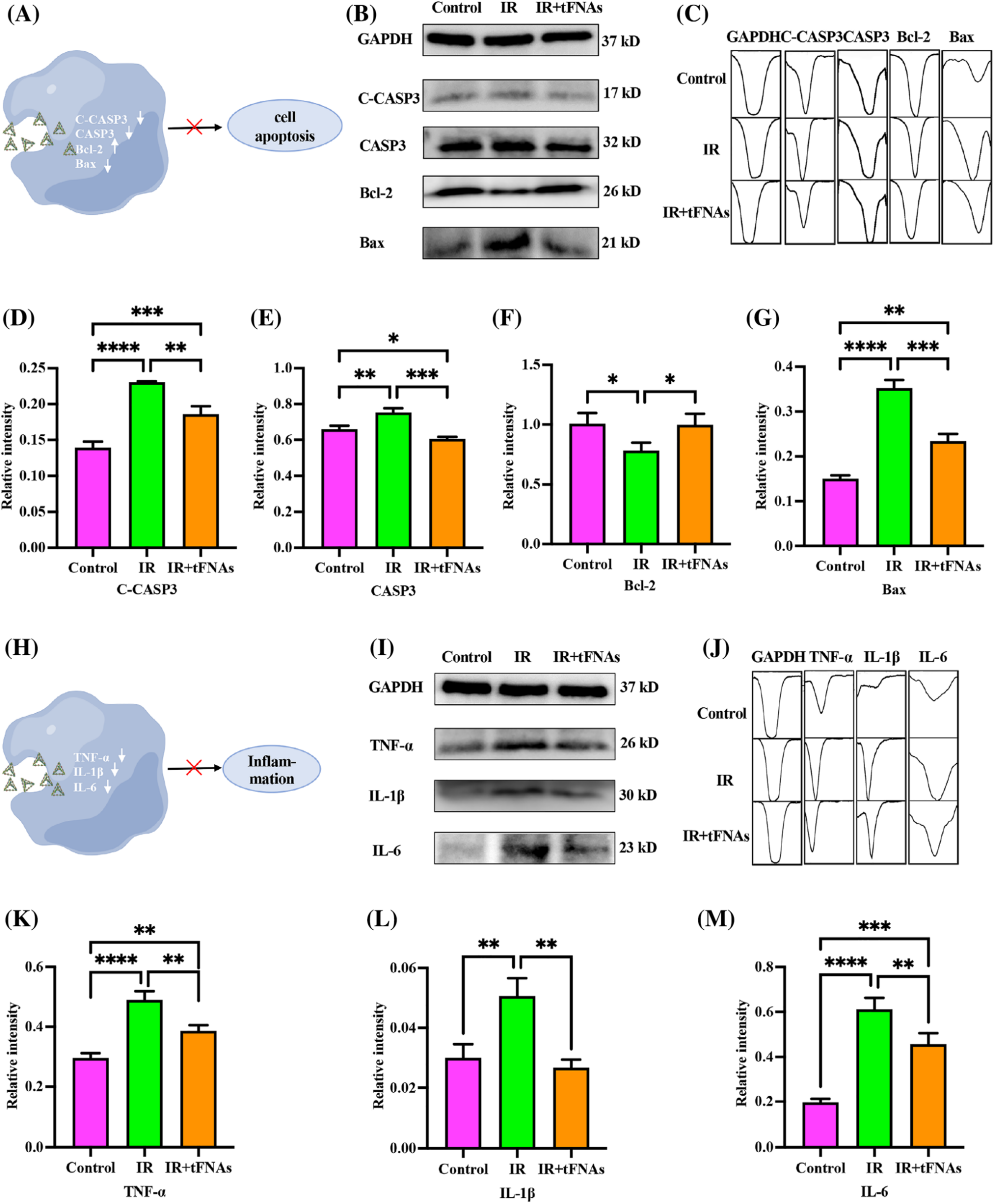
Expression of apoptotic proteins and inflammatory factors. (A) The schematic diagram of the relationship between expression of C‐Caspase 3, Caspase 3, Bcl‐2 and Bax and apoptosis. (B) The expression of C‐Caspase 3, Caspase 3, Bcl‐2 and Bax measured by western blot. (C) Histogram of protein expression analysed by Image J. (D–G) Quantitative analysis of C‐Caspase 3, Caspase 3, Bcl‐2 and Bax expression level. (H) The schematic diagram of the relationship between expression of TNF‐α, IL‐1β and IL‐6 and inflammation. (I) The expression of TNF‐α, IL‐1β and IL‐6 measured by western blot. (J) Histogram of cytokine expression analysed by Image J. (K–M). Quantitative analysis of TNF‐α, IL‐1β and IL‐6 expression level. Data were presented as mean ± SD, *n* = 3, one‐way ANOVA and Tukey multiple comparisons was used to calculate *p*‐value, *p < 0.05, **p < 0.01, ***p < 0.001, ****p < 0.0001.

The above experimental results showed that tFNAs could inhibit apoptosis in irradiation‐damaged SMGCs. It is widely known that Caspase and Bcl‐2 are widely concerned apoptotic protein families.^38^ Among them, Bcl‐2 and Bax are the most important regulatory proteins whose functions are opposite to each other, that is, high expression of Bax promotes apoptosis and high expression of Bcl‐2 inhibits apoptosis.^39^, ^40^ Caspase3 is the most critical apoptotic executive protease in the process of apoptosis^41^ (Figure 3A). We detected the expression of the Bcl‐2/Bax/Caspase‐3 signalling pathway via western blotting. After SMGCs were irradiated and incubated with 125 nM tFNAs for 48 h, the total cell protein in each group was extracted. The expression of C‐Caspase 3, Caspase3 and Bax was upregulated, and Bcl‐2 downregulated by irradiation, after tFNAs treatment the protein expression was reversed (Figure 3B). Histogram of protein expression analysed by Image J was showed in Figure 3C. The expression of C‐Caspase 3, Caspase3 and Bax in the tFNAs‐treated group was 1.26‐fold, 1.16‐fold and 1.51‐fold lower than that in the irradiation only group (*p* < 0.01, *p* < 0.001 and *p* < 0.001; Figure 3D,E,G); the expression of Bcl‐2 was 1.27‐fold higher after tFNAs treatment than that in the irradiation only group (*p* < 0.05; Figure 3F). Based on these results, tFNAs inhibited apoptosis of irradiation‐damaged SMGCs through regulating the Bcl‐2/Bax/Caspase‐3 signalling pathway.

### 3.8 tFNAs inhibit the release of inflammatory cytokines

According to previous reports, irradiation‐induced salivary gland damage is attributed to elevated levels of oxygen free radicals induced by irradiation. Increased oxygen free radicals could cause abnormal inflammatory responses.^42^ TNF‐α, IL‐1β and IL‐6 are very important proinflammatory cytokines with a range of biological activities^43^ (Figure 3H). We detected the expression of TNF‐α, IL‐1β and IL‐6 via western blotting. The expression of TNF‐α, IL‐1β and IL‐6 was upregulated by irradiation and downregulated after tFNAs treatment (Figure 3I). Histogram of these cytokines expression analysed by Image J were showed in Figure 3J. When compared with the irradiation‐only group, the expression of TNF‐α in the tFNAs‐treated group was decreased 1.27 folds (*p* < 0.01; Figure 3K); the expression of IL‐1β in the tFNAs‐treated group was decreased ~1.89 folds (*p* < 0.01; Figure 3L); the expression of IL‐6 in the tFNAs‐treated group was decreased about 1.34 folds (*p* < 0.01; Figure 3M). These results revealed that tFNAs could inhibit the release of inflammatory factors induced by irradiation and thus could reduce cell damage.

### 3.9 Functional improvement after tFNAs treatment

**FIGURE 4:**
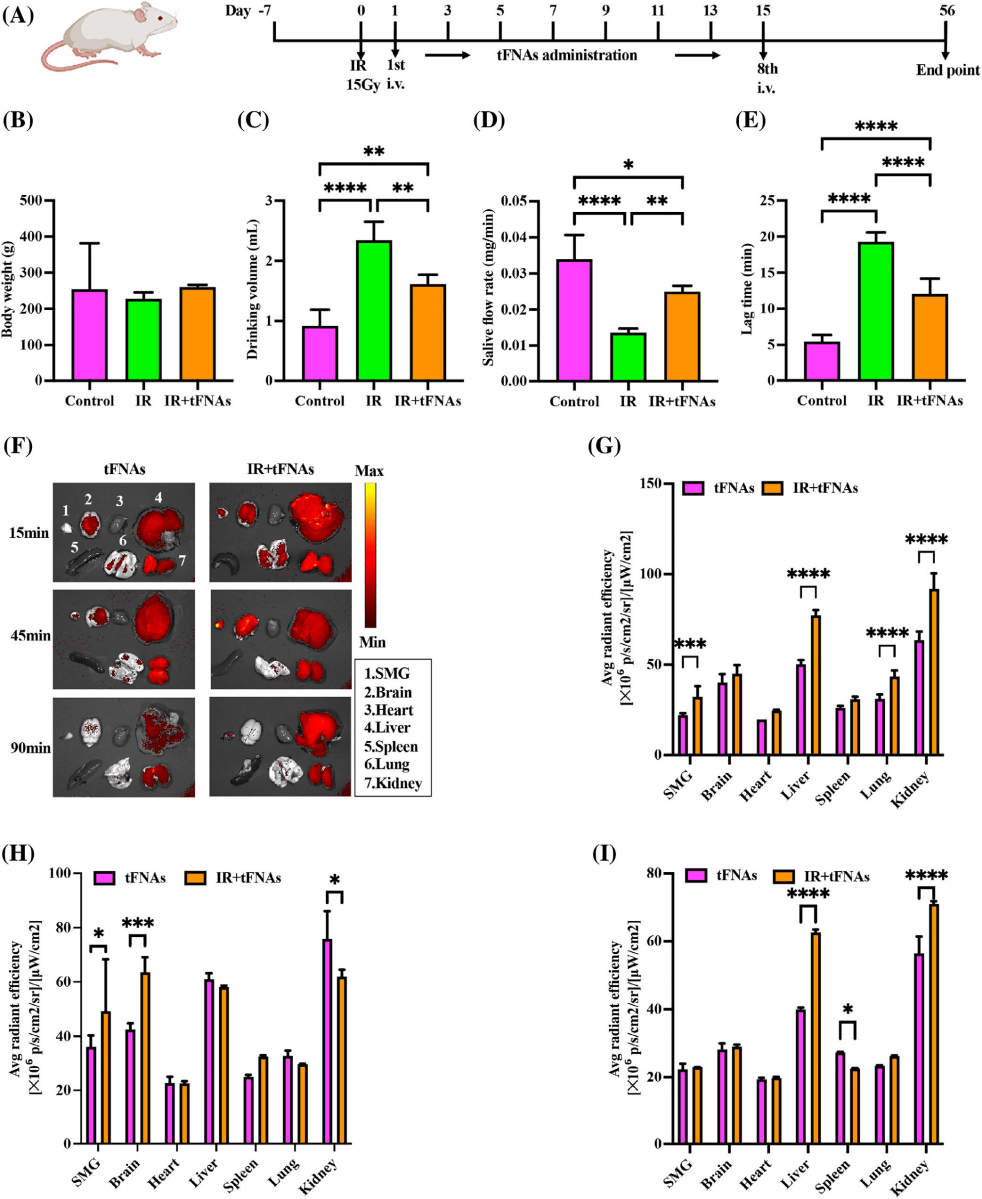
Functional outcoming of submandibular gland (SMG) and biodistribution of tetrahedral framework nucleic acids (tFNAs). (A) Schematic of experimental design. (B) Body weights. (C) Drinking volume. (D) Saliva flow rate. (E) Lag time. (F) Fluorescence images of tFNAs biodistribution in major organs and SMG. (G–I). Fluorescence intensity analysis of each organ at 15, 45 and 90 min post‐injection. Data were presented as mean ± SD, *n* = 3, one‐way ANOVA and Tukey multiple comparisons was used to calculate *p*‐value in (B–E), two‐way ANOVA and Tukey multiple comparisons was used to calculate *p*‐value in (G–I), *p < 0.05, **p < 0.01, ***p < 0.001, ****p < 0.0001.

Schematic of experimental design was shown in Figure 4A. At Week 8 after irradiation, there was no significant difference in body weight between all groups (Figure 4B). The water intake in each group was also measured at Week 8 after irradiation. Irradiation significantly increased the water intake of PBS‐injected group compared with normal group (2.55‐fold), and tFNAs administration appeared to significantly reduce water intake as compared with PBS‐injected group (0.69‐fold, *p* < 0.01; Figure 4C). These results showed that tFNAs could alleviate the increase of water intake caused by irradiation‐induced SMG damage.

At Week 8, we measured saliva production and salivation lag time to evaluate whether the salivary function was improved after tFNAs administration. SFR in PBS‐injected group was 0.4‐fold lower than that in normal group (*p* < 0.01), while after tFNAs treatment, saliva production was significantly increased 1.83‐fold compared with PBS‐injected group (*p* < 0.05) and has no difference from the normal group (Figure 4D). After SMGs were irradiated, salivation lag time was much longer in PBS‐injected group than the lag time in normal group (3.55‐fold). After tFNAs treatment, lag time was reduced 0.63‐fold compared with PBS‐injected group, which was significant (*p* < .05, Figure 4E).

### 3.10 tFNAs effectively accumulated in the SMG after irradiation

tFNAs was intravenously injected into normal and irradiated SD rats. 15, 45 and 90 min post‐injection, we performed ex vivo imaging of major organs (brain, heart, liver, spleen, lung and kidney) and SMG. The result showed that strong fluorescence was detected in the SMGs of the irradiated rats (Figure 4F). At 15 and 45 min, fluorescence intensity of SMGs of normal and irradiated rats was significantly different (*p* < 0.001 and *p* < 0.05; Figure 4G,H), and there was no difference at 90 min as the metabolism gone on (Figure 4I). Therefore, tFNAs could effectively accumulated in the SMG after irradiation, which might be responsible for function improvement after tFNAs treatment. This phenomenon might be attributed to the increased permeability caused by tissue damage. Notable fluorescence was observed in the liver and kidney, which indicated that tFNAs might be metabolized by the liver and excreted by the kidney.

### 3.11 Micromorphological changes

**FIGURE 5:**
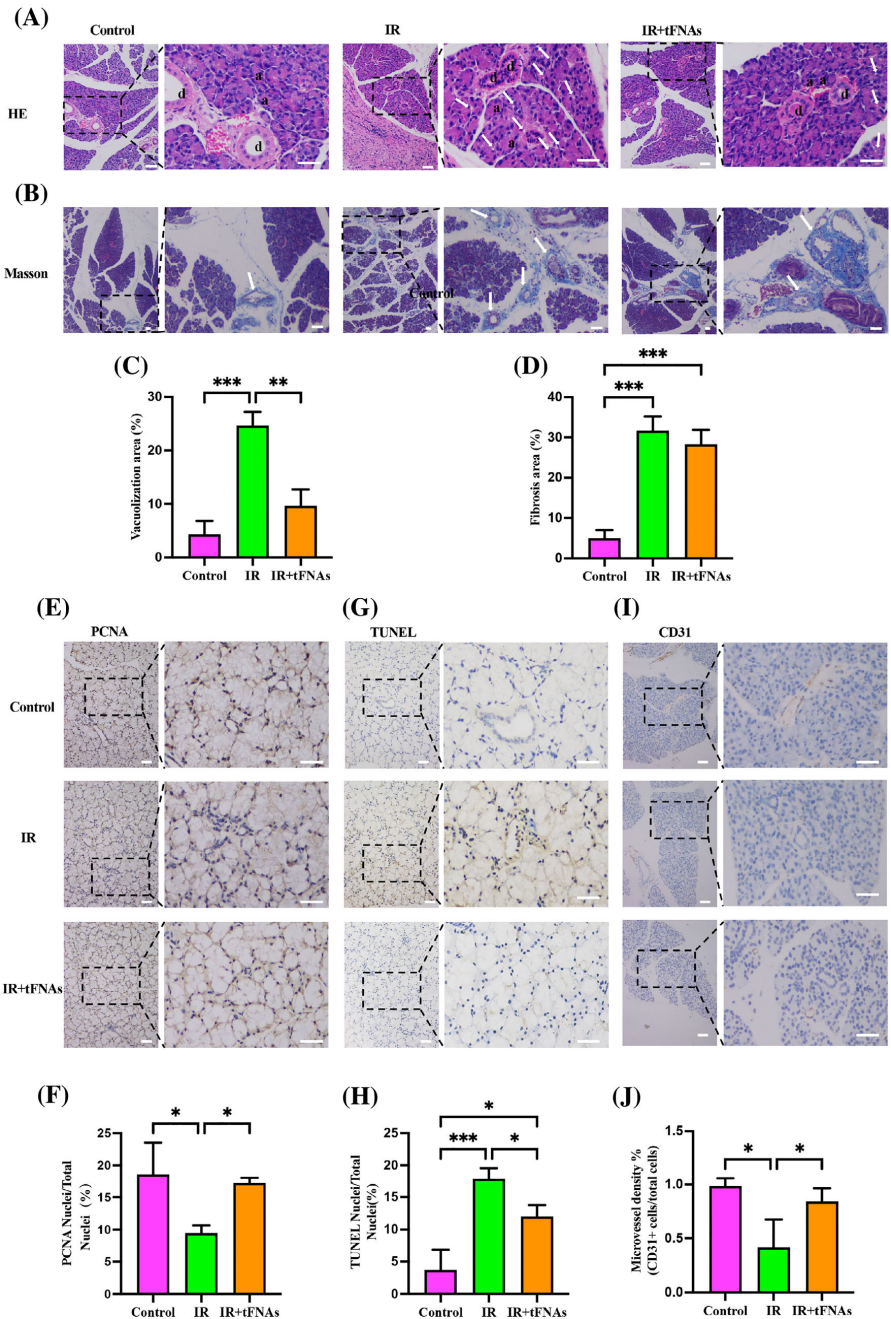
Protected effect of tetrahedral framework nucleic acids (tFNAs) against irradiation‐induced submandibular gland (SMG) damage. (A) haematoxylin–eosin stains. Arrows indicate vacuolization. “a” and “d” indicate acinar cells and ductal cells, respectively. (B) Masson staining. Arrows indicate fibrosis. (C) Statistical analysis of vacuolization area. (D) Statistical analysis of fibrosis area. (E) Immunofluorescence staining for proliferating cell nuclear antigen (PCNA). (F) Quantification of PCNA‐positive proliferative cells. (G) TUNEL assay. (H) Quantification of TUNEL‐positive apoptotic cells. (I) Immunofluorescence staining for CD31. (J) Microvessel density was quantified by the ratio of CD31‐positive cells to total cells. Scale bar = 50 μm. Data were presented as mean ± SD, *n* = 3, one‐way ANOVA and Tukey multiple comparisons was used to calculate *p*‐value, *p < 0.05, **p < 0.01, ***p < 0.001.

Micromorphological changes including structural changes and fibrosis were visualized by HE staining and Masson staining, respectively. In normal SMGs, the acinar cells were full, and the glandular lobules were clear. In PBS‐injected SMGs, acinar cells were atrophied, vacuolation degeneration and cytoplasm was deeply stained. The acinar structure was recovered in tFNAs‐treated SMGs (Figure 5A). Statistical analysis of vacuolization area indicated that tFNAs treatment could significantly alleviate irradiation‐induced acinar damage (Figure 5C). After Masson staining, the fibrous tissue appeared blue. Mild fibrosis was observed in PBS‐treated SMGs, in a concentric circle shape (Figure 5B). The difference in the degree of fibrosis between tFNAs‐treated and PBS‐injected SMGs was not significant (Figure 5D).

### 3.12 tFNAs protect salivary gland from irradiation‐induced damage

These results suggested that tFNAs could improve the morphology and function of irradiation‐damaged salivary gland. Next, we explored the mechanisms of these phenomenon. The proliferative potential of SMGs was evaluated by immunohistochemistry of PCNA. PCNA was expressed in the nucleus and stained brownish yellow. Immunohistochemistry of PCNA at Week 8 revealed that more PCNA‐positive proliferating cells were found in tFNAs‐treated SMGs than PBS‐injected SMGs (Figure 5E). Semi‐quantitative analysis of the immunohistochemical images showed that the PCNA‐positive proliferating cells in tFNAs‐treated SMGs were 1.43‐fold higher than those in PBS‐injected SMGs, which was significant (*p* < 0.05; Figure 5F).

SMG apoptosis was detected via TUNEL staining. To distinguish apoptotic cells from normal cells, apoptotic cell nuclei were stained dark brown using the DAB chromogenic method, whereas normal cell nuclei were stained blue with haematoxylin re‐staining. The tFNAs‐treated SMGs showed fewer TUNEL‐positive apoptotic cells than PBS‐injected SMGs (Figure 5G). Statistical analysis was shown in Figure 5H. The difference in TUNEL‐positive apoptotic cells between the tFNAs‐treated SMGs and PBS‐injected SMGs was significant (0.67‐fold; *p* < 0.05).

In recent years, studies have shown that microvascular endothelial cells in salivary glands are important targets of irradiation injury. Protecting the microvascular system may prevent irradiation‐induced salivary gland hypofunction and allow acinar progenitor cells to recover from initial injury.^19^ To investigate whether tFNAs administration could protect the microvascular system in SMGs from irradiation damage, we tested the expression of CD31 by immunostaining. The expression of CD31 was decreased in PBS‐injected SMGs and increased after tFNAs treatment (Figure 5I). Microvessel density was calculated as the ratio of CD31‐positive cells to total cells, which was significantly increased in tFNAs‐treated SMGs compared with PBS‐injected SMGs (1.98‐fold; *p* < 0.05; Figure 5J). Therefore, the microvascular system in irradiation‐damaged SMG was benefit from tFNAs treatment.

## 4 CONCLUSIONS

In this study, we found that tFNAs treatment could promote cell proliferation and inhibit cell apoptosis of irradiation‐ damaged SMGCs and reduce irradiation induced cell death. Mechanism studies revealed that tFNAs inhibited cell apoptosis through regulating the Bcl‐2/Bax/Caspase‐3 signalling pathway and inhibited the release of TNF‐α, IL‐1β and IL‐6 to reduce cell damage caused by inflammation. Animal experiments demonstrated that tFNAs administration could improve salivary function and reduce acinar cell damage after irradiation. Ex vivo imaging of major organs and SMG confirmed that tFNAs could accumulated more efficiently in the SMG of irradiated rats, which might be attributed to the positive effects on irradiation‐damaged SMG. The results of PCNA and TUNEL immunostaining suggested that tFNAs could increase tissue regenerative activity and reduce apoptosis. In addition, we found that tFNAs could alleviate microvascular injury caused by irradiation and had an obvious vascular protective effect. These findings were consistent with previous reports of tFNAs showing tissue regenerative activity, such as promoting cell proliferation and blood vessel formation, and anti‐apoptosis.^44^ Based on these results, we believe that tFNAs could serve as a promising agent to protect salivary glands from irradiation‐induced damage.

## AUTHOR CONTRIBUTIONS

Xueping Xie, Wenjuan Ma and Yunfeng Lin involved in conceptualization; Xueping Xie, Wenjuan Ma and Yuxi Zhan involved in data curation; Xueping Xie and Wenjuan Ma formally analysed; Guo Li and Yunfeng Lin involved in funding acquisition; Xueping Xie, Wenjuan Ma and Guo Li involved in methodology; Yunfeng Lin administrated the project; Xueping Xie wrote the original draft; Xueping Xie, Wenjuan Ma and Yuxi Zhan reviewed and edited the writing. All authors read and approved the final article.

## CONFLICT OF INTEREST

The authors declare no competing financial interests.

## Data Availability

The data that support the findings of this study are available from the corresponding author upon reasonable request.
